# What is the impact of forced displacement on health? A scoping review

**DOI:** 10.1093/heapol/czad002

**Published:** 2023-01-11

**Authors:** Cristóbal Cuadrado, Matías Libuy, Rodrigo Moreno-Serra

**Affiliations:** Ministry of Health of Chile, Enrique Mac Iver 541, Santiago, Chile; Escuela de Salud Pública, Universidad de Chile, Av. Independencia 939, Independencia, Santiago, Chile; Centre for Health Economics, University of York, Alcuin A Block, Heslington, York YO10 5DD, UK

**Keywords:** Displaced populations, immigrants, health outcomes, econometrics, impact, reproductive health, research methods

## Abstract

While there is a broad literature analysing the effects of migration on health, important knowledge gaps persist particularly on the causal effects of forced displacement on health outcomes. We undertake a scoping review of applied epidemiological, statistical and econometric studies examining causal health impacts of forced displacement, which initially identified 1454 studies from the health and social sciences disciplines published up to May 2021. Our study makes two key contributions. First, we offer a comprehensive overview of the evidence generated, methodologies adopted and analytical challenges faced by current research examining the causal relationship between forced displacement and health. Second, we present concrete examples of how key challenges around study design and estimation approaches influence the strength of the evidence-base on the topic, using as a case study the broad domain of reproductive health. We find that, beyond the increased mortality risk that can be attributed to forced displacement, most of the available empirical evidence for a wide range of health outcomes is prone to substantial bias, making it difficult to draw firm conclusions. Our synthesis of credible studies conducted in different settings indicates that current research practice in the field could be strengthened through selection of valid control groups and application of more appropriate causal inference methods. Our findings are useful to promote the generation of further evidence on the topic that can reliably inform the design of policies to protect the health of displaced populations.

Key messages

- We offer a comprehensive overview of the analytical challenges faced by current research from the social and health sciences, examining the relationship between forced displacement and health, as well as of the main methodological approaches employed to potentially address those challenges.
- We present examples of how these challenges influence the strength of the evidence-base on the topic, using as a case study the broad domain of reproductive health, including maternal, perinatal and child health.
- We find that, beyond the effects of increased mortality risk that can be attributed to forced displacement, most of the available empirical analyses for a wide range of health outcomes report findings that are prone to substantial bias, making it difficult to draw firm conclusions. This is due to issues around selection of valid control groups and the application of credible causal inference methods in several studies.
- Based on our findings, we offer guidance on how current research practice in the field could be strengthened, and also guide the design of appropriate policies that may protect the health of displaced populations.

## Introduction

Migration is a growing and widespread phenomenon in our society. It involves any movements of individuals from their usual place of residence to another, within or outside their home country. One of the most prominent aspects of globalization in the last decades has been the substantial increase in human mobility. It is estimated that 272 million individuals are international migrants and 740 million are internal migrants within their own country (IOM, 2020). Forced displacement represents a major driver of global migration currently. In 2020, 55 million people were internally displaced, with 40.5 million new displacements occurring during that year (Internal Displacement Monitoring Centre (IDMC), 2021). Most people who were forcibly displaced during the last decade are in Africa, Latin America and Asia, where a very important growth year-on-year of numbers displaced has taken place over the last decade, with no sign of reduction. Globally, 82.4 million people were displaced due to conflicts, violence or natural disasters in 2020 (UNHCR, 2021).

Standard terminology used to refer to individuals according to their different migration status or contexts (international migrants, internal migrants, displaced persons, refugees and asylum seekers) is outlined in Table 1. In this article, we focus on forced displacement, as it represents a global humanitarian issue that has become a key driver of migration flows, as indicated above. Forced displacement occurs when individuals are compelled to relocate from their places of usual residence due to disasters, be they natural (e.g. storms, floods, droughts, earthquakes) or human-made (e.g. armed conflict or violence). Such processes can lead to internal (within-country) or international (across-borders) migration.

**Table 1.** Definitions of migration, forced displacement and related concepts

International migrants	Individuals who remain outside their usual country of residence for at least 1 year.
Internal migrants	Individuals who move within the borders of a country, usually measured across regional, district or municipal boundaries, resulting in a change of usual place of residence.
Displaced persons	Individuals who have been forced to leave their homes or places of usual residence, in particular because of, or to avoid, the effects of armed conflict, situations of generalized violence, violations of human rights, or natural or man-made disasters. In cases when they have not crossed an international border, they are referred to as internally displaced persons (IDPs).
Refugees	Individuals who, owing to a well-founded fear of being persecuted for reasons of race, religion, nationality, membership of a particular social group, or political opinion, are outside the country of their nationality, and are unable to or, owing to such fear, unwilling to avail themselves of the protection of that country or return because of fear of persecution.
Asylum-seekers	Individuals who have sought international protection and whose claims for refugee status have not yet been determined.

Source: adapted from Zimmerman *et al*. (2011).

Despite the global importance of forced displacement flows, there are important gaps in the current knowledge base about this phenomenon. Two inter-related gaps are particularly salient. First, while there is some literature analysing the effects of forced migration on health, there is a lack of studies offering a comprehensive synthesis of the evidence produced so far for different health domains and from different disciplinary perspectives. Second, for the evidence that has been produced on the topic, it is unclear how reliable the conclusions drawn from these studies can be considered to be, given the typical methodological and data challenges faced by research in this field. We discuss each of these two gaps in the literature—synthesis of the available evidence about the health impacts of forced displacement, and overall assessment of the reliability of such evidence—in what follows.

Health can be expected to represent a particularly vulnerable domain among displaced populations. The available evidence on the impact of displacement on health focuses on a wide range of areas including mental health (Virgincar *et al.*, 2016; Vossoughi *et al.*, 2016; Turrini *et al.*, 2017), alcohol consumption (Weaver and Roberts, 2010), perinatal outcomes (Heslehurst *et al.*, 2018) and oral health (Keboa *et al.*, 2016). Additionally, some evidence is available associating forced displacement with the transmission of antimicrobial resistance (Willem *et al.*, 2017). Several pathways can explain these findings. Traumatic life events have been shown to produce direct effects on health outcomes (Bauer *et al*., 2019). There is also credible evidence about the long-term effect of stress due to the displacement increasing the likelihood of ischaemic heart disease (Haukka *et al.*, 2017). Other effects can be explained through poor integration of displaced populations into the host society (e.g. marginalization and lower socioeconomic status than native individuals), producing a ‘cumulative disadvantage’ that leads to negative effects on health outcomes (Bauer *et al.*, 2019). In the short-term, before resettlement, displaced populations tend to live in poor sanitary conditions, with inadequate shelter, affected by crowding and suboptimal nutrition. All of these factors are linked to increased risk of communicable diseases (Banner *et al.*, 2020). These short- and long-term negative health consequences of forced displacement are likely to be compounded by the high prevalence of exposure to different types of violence (Mollica *et al.*, 2001), adverse labour market outcomes and heightened risk of poverty (Becker and Ferrara, 2019; Verme and Schuettler, 2021) among displaced populations.

Previous studies have reviewed the analytical (statistical and econometric) quantitative methods and the evidence generated by studies assessing various impacts of forced displacement on migrants and host communities (Ruiz and Vargas-Silva, 2013; Becker and Ferrara, 2019; Verme and Schuettler, 2021). Nevertheless, these evidence review studies have mainly taken a labour economic lens by looking at employment, income, prices, consumption and production as main outcomes. By contrast, little attention has been given by previous review studies to impacts on health outcomes. As an example, Becker and Ferrara (2019) include only three studies reporting health consequences of forced displacement on migrants. In the health sciences literature, most existing analyses and evidence syntheses are non-comparative observational studies that report descriptive statistics (e.g. prevalence of a certain condition) or simple differences of displaced vs non-displaced populations (either host or home-populations). The comparison groups are often very heterogeneous, producing sometimes contradictory results or findings that are difficult to reconcile. Overall, there is a lack of evidence review studies that employ a systematic approach to assess if associations between displacement and health outcomes in the literature can be considered as causal. It is also noteworthy that the inclusion of literature of either health or social sciences (depending on the disciplinary audience), but not from both disciplines, has been the norm of previous reviews in the field. This common limitation hampers the complementary learnings that can be drawn from studies examining very similar questions but from different disciplinary and methodological lenses. It also highlights the need for an integrated analysis of the body of available evidence.

This paper makes two contributions to the literature. First, we review a large body of literature from both the health and social sciences disciplines to provide a comprehensive overview of the evidence generated, methods adopted and analytical challenges faced by current research examining the relationship between forced displacement and health. We are particularly interested in the extent to which the methodological approaches adopted have overcome (or not) key analytical challenges for applied research in the field, thereby producing evidence that can be interpreted as ‘causal’ links running from forced displacement to health, including the pathways through which forced displacement can impact population health. Reliable identification of these links is not trivial due to the mostly observational nature of the data available and the difficulties in identifying adequate control groups (see e.g. the studies reviewed by Becker and Ferrara, 2019). This creates important challenges to arrive at causal conclusions on the impacts and key mechanisms through which displacement influences health.

Second, we zoom in further on the results of our scoping review to provide a detailed illustration of the implications of these challenges for the existing knowledge base about the impacts of displacement on broad dimensions of reproductive health (including maternal, perinatal and child health). We also discuss the methodological approaches selected by these studies focusing on reproductive health to address the challenges for establishing causal links between displacement and health. Assessing the robustness of the available evidence about the health impacts of forced displacement, as well as offering guidance about how this evidence could be strengthened, are areas that must be urgently addressed given the large scale of forced displacement globally, to take stock of what we actually know about the topic. Notwithstanding the substantial methodological difficulties, the generation of reliable causal inference about the links between displacement and health is important not only from an academic knowledge viewpoint. It is also crucial for guiding the design of appropriate policies that can protect the health of displaced populations.

The article is organized in four sections in addition to this introduction. The Methods section presents the methodologies for our scoping literature review about forced displacement and health and for categorizing the strength of the available evidence in the field. The Results section discusses the main findings from our scoping review, synthesizing the key analytical challenges that we identify for applied studies in the field, including those pertaining to data, study design and quantitative methodology. This section also summarizes the main methodological approaches that studies have adopted to address such challenges and disentangle the causal relationship between forced displacement and health. In the Case study, we present specific examples of how the key analytical challenges identified influence the strength of the evidence-base in the area, using as a case study the broad domain of reproductive health. Finally, the Discussion and conclusions section discusses our findings, offering methodological guidance on how current research practice in the field could be strengthened, which we argue is useful for applied health and social science researchers conducting studies on migration more generally.

## Methods

We conducted a scoping literature review to identify the main econometric and epidemiological methods for causal inference currently applied to causal questions involving forced displacement as the exposure (‘treatment’) variable and any health outcome. We considered a scoping review to be appropriate in our context due to its ability to capture different types of studies, and in particular, for facilitating a comprehensive synthesis of the available literature on a topic that has been studied across multiple disciplines with differing outcomes of interest and methodological approaches, such as the health consequences of displacement. We follow the Preferred Reporting Items for Systematic reviews and Meta-Analysis extension for Scoping Reviews (PRISMA-ScR) reporting guidelines; the PRISMA-ScR checklist is provided in the online supplementary material (SM5).

### Review questions, data sources and search strategy

Our review sought to investigate the following question: what are the analytical challenges faced by current quantitative research examining the relationship between forced displacement and health, as well as the main methodological approaches employed to address those challenges? A key objective of the review was to serve as a basis for our systematic assessment of the quality of the available evidence on the causal links between forced displacement and health outcomes.

We searched Epistemonikos, the largest database of systematic reviews in health, that on 10 April 2021 included 368 071 systematic reviews. This database is maintained by screening multiple information sources, including MEDLINE, EMBASE, Cochrane, CINAHl, PsycINFOm LILACS, The Campbell Collaboration, JBI Database of Systematic Reviews and Implementation Reports, among others, to identify systematic reviews and their included primary studies. A detailed overview of this database is available elsewhere (Epistemonikos Foundation, 2021). Additionally, we conducted search strategies of primary studies in the EconLit database, to widen the scope towards the economic literature, and EMBASE, to ensure completeness of the inclusion of relevant primary studies from the health sciences. We used the following search terms for Econlit: (refugee OR refugees OR asylum OR displace* OR (forced AND migra*)) AND health; and EMBASE: (‘refugee‘/exp OR ‘asylum‘/exp OR ’protracted displacement’ OR ‘internally displaced persons’/exp) AND health AND caus*.

Studies included were limited to May 2021 without language limits. Additional articles were identified through the different references found in other reviews or systematic reviews of interest. For working papers, pre-print and other pre-publication records, the latest version of the article was manually searched to increase completeness of the information included in the analysis. The full electronic search strategy can be found in the online supplementary material (SM1).

### Study selection and data extraction

We identified 1454 records from the databases above. After collecting all references, searches were merged and duplicates removed, followed by screening of the titles and abstracts. The inclusion criteria were: (1) studies including any forced displaced population; and (2) comparative studies that attempt to make causal claims on the effect of forced displacement on any health-related outcome. The exclusion criteria were: (1) studies where the migration status (forced or voluntary) of the population under study was unclear; (2) no full-text available; and (3) studies analysing effects of conflict or other emergencies without explicitly addressing displacement. The full text was reviewed, considering the inclusion and exclusion criteria. If one study had multiple reports, they were linked and considered as a single record. Two researchers screened abstracts and reviewed the full texts. All the references were managed using Mendeley 1.19.8. The complete PRISMA flow diagram with the number of records included and excluded in each step of the review are presented in the online supplementary material (SM2).

After screening and reading of the full articles, when available, we extracted data from the reviews identified, reanalysed data from primary studies included in those reviews and added other relevant primary studies not included by previous systematic reviews. A data extraction form was created that included the following information: author, year, reference, characteristics of the displaced population (host setting, setting of origin, conflict or disaster event), data collection period, outcomes of interest (including access to health services, health expenditure and financial-risk protection, all-cause mortality, cause-specific mortality, disease incidence and equity in those outcomes), data (sources, type of data—individual vs aggregated, cross-sectional vs longitudinal) and details about the quantitative identification strategy (comparator group, study design, assumptions). The data extraction form is available in the online supplementary material (SM3).

### Quality appraisal

We follow previous work (Channa and Faguet, 2016) and classify the quality of the evidence according to a four-level categorization: very strongly credible, strongly credible, somewhat credible and less credible. This classification relies on the capacity of the paper’s methodological (identification) strategy to produce a valid comparison group and to mitigate endogeneity concerns^1^. Type of studies and justification for each category are provided in Table 2.

**Table 2.** Quality rating for the evidence

Quality of evidence	Type of study	Justification
Very strongly credible	Randomized control trials(RCTs)	‘Gold standard’ for identifying causal effects.
Strongly credible	Quasi-experimental techniques such as natural experiments, instrumental variables (IV), difference-in-differences (DID) approaches, regression discontinuity design (RD), interrupted time-series (ITS) or high-quality panel data estimations using fixed effects.	Reasonably successful in producing a valid comparison group with limited endogeneity in which main assumptions are adequately fulfilled.
Somewhat credible		Studies less likely to produce a valid comparison group, with substantial endogeneity or in which main assumptions do not hold (e.g. parallel trends in a DID).
Less credible	Simple comparisons (e.g. unadjusted differences between groups) or regression methods with observational data that fail to control for endogeneity bias.	Observational studies that are unable to produce a valid comparison group.

Source: adapted from Channa and Faguet *et al*. (2016).

### Data synthesis

We conducted a qualitative synthesis of the data collected, describing the empirical strategies adopted across all reviewed studies based on comparison groups, type of data and study design adopted^2^. We summarize the main findings from our qualitative synthesis in the next section, focusing on the main analytical challenges for applied studies in the field, as well as the key issues identified around selection of comparison groups, main health outcomes examined and estimation approaches. Based on these findings, in the next section we also propose a taxonomy of possible comparison groups for empirical research in the area, discuss the strengths and limitations of these methodological approaches, highlight their main assumptions and offer recommendations about study design.

Finally, for the case study, we present a summary and discussion of our findings from the qualitative synthesis (as detailed above), specifically for the reviewed studies that focused on the effects of forced displacement on reproductive health outcomes. We focus on the latter set of outcomes as a case study due to the heightened sexual and reproductive health risks typically faced by women in situations of forced displacement (including the risks of sexually transmitted diseases, maternal/child morbidity and mortality) and the resulting acute need for evidence that can inform policies to protect the sexual and reproductive health of displaced women and girls during their migration journey and in the host communities (Starrs *et al.*, 2018).

## Results

### What are the main analytical challenges for applied studies in the field?

#### (Non)random allocation

A common starting point is the assumption that displacement events are exogenous^3^ and, therefore, differences between displaced and non-displaced populations can be interpreted as causal effects. This is more likely to hold in contexts where the entire (clearly identified) population in a particular territory is homogenously exposed at the same time, e.g. in Ceded Karelia, Finland, during World War II (Saarela and Finnas, 2009; Saarela and Elo, 2016). During World War II, the entire population of the then-Finnish Ceded Karelia region was forcibly evacuated and, after the war, Karelians could not return home because the area was ceded to the Soviet Union. This exogenous event generated a large group of forcibly displaced Finns for whom individual characteristics can be considered to be unrelated to displacement from the home territory, thus favouring direct comparisons of health outcomes between displaced Finns and non-displaced populations (e.g. people born on the adjacent side of the new border; Saarela and Elo, 2016). In such a context, exposure to displacement can be considered as good as random, which is often referred to as a natural experiment setting. However, although the randomness of displacement exposure in a natural experiment setting (as in Saarela and Elo, 2016) favours the causal attribution of health differences between exposed and non-exposed populations to the impact of displacement, this may not be enough to ensure non-biased estimations of displacement impacts. This would be the case if there remain other observable or unobservable differences between exposed and non-exposed populations under comparison that may influence the health outcomes being evaluated. Thus, even in such natural experiment settings, it is often important to employ analytical models that can account for the influence of further potential observable and unobservable differences between populations in the specific evaluation context, such as difference-in-differences or interrupted time series approaches, which we discuss further below (to this end, Saarela and Elo (2016) estimate discrete time hazard models with data on individual- and area-level covariates for years between 1988–2012).

Nevertheless, in most situations of war, conflict or natural disasters, there is some degree of agency in the decision to (not) migrate—and, therefore, no randomness in exposure to displacement. Who migrates (because they want or have to, and have the resources for moving), when and where are all influenced by individual ‘self-selection’, potentially introducing selection bias for empirical analyses through non-random allocation of the exposure of interest. This is particularly relevant when the variable of interest (e.g. a health outcome) is also related to the likelihood of exposure. Limited ability to account for the potential endogeneity introduced by individual characteristics that may influence both displacement status and health outcomes has hampered the reliability of the evidence generated by some applied studies, e.g. the analysis of conflict-induced displacement effects on the mortality of children <5 years old in Uganda and Sudan (Singh *et al.*, 2004) and of displacement impacts on pregnancy outcomes among Somali women (Small *et al.*, 2008). Moreover, the intensity of events like conflict violence is not always homogeneous, with some conflicts affecting disproportionally certain individuals within a community, for instance specific ethnic groups. This phenomenon, known as targeting, can be based on characteristics not observed by the researcher, hence imposing threats to reliable inference (e.g. for the examination of the impacts of displacement induced by the Balkans War on pregnancy outcomes among women from different ethnic backgrounds in Croatia, Serbia, and Bosnia and Herzegovina; Kuvacic *et al.*, 1996).

#### Limited comparability between displacement contexts

For analyses of the impact of forced displacement on health, it is important to recognize that the exposure of interest is heterogeneous and heavily determined by contextual factors. For example, we could expect different exposure and potential health consequences of a state-organized mass-displacement to neighbour territories with governmental support (e.g. Ceded Karelia or East Germany during World War II; e.g. Saarela and Elo, 2016), compared to the displacement of populations in protracted civil conflict areas in low-income countries (e.g. the Darfur conflict or the Syrian civil war; e.g. Erenel *et al.*, 2017), or where migration outside the conflict area is unfeasible (e.g. West Bank; Mansour and Rees, 2012). The type of events producing displacement could range from acute shocks (e.g. earthquakes) to protracted conflicts, with varying degrees of intensity and spread over time. Accounting for such contextual characteristics is key for the design of an adequate empirical strategy, but at the same time these contextual differences may limit the interpretability and comparability of findings in the literature more generally.

A related issue is the selection of migration destination. This is influenced by the context of the conflict or emergency, such as geographical and political factors, and individual characteristics that enable individuals to migrate. Host territory characteristics can influence long-term outcomes in different ways. Situations during the displacement and relocation process, such as requirements for legal recognition, prosecution of undocumented migrants, access to assistance programmes and opportunities for integration, can mediate the long-term health effects on forcibly displaced populations. Additionally, the decision to migrate to certain territories could be determined by some of these very same host-territory characteristics (e.g. better salaries or employment opportunities). Therefore, different population groups displaced by the same event can experience different health consequences, not only due to their varying degrees of exposure to the event within the home territory, but also due to systematically different experiences during the relocation process and arrival in the host territory. Just like a year of exposure to an armed conflict in a particular context may not be the same as in another context, how a displacement event is experienced by one group is not necessarily comparable to the experience of another group. This hampers the potential to generalize conclusions obtained from existing comparative analyses [such as Hynes *et al*.’s (2012) study on maternal mortality among displaced populations in 10 countries]. Little attention has been paid to this point in the literature, which is as relevant for within-study estimation of average treatment effects as it is for data synthesis in meta-analysis.

#### Disentangling the effects of violence from the impact of forced displacement

The exposure or ‘treatment’ of interest is not always adequately specified across studies. Since forced displacement is linked to events like natural or man-made disasters, in some cases it is not clear what the main phenomenon under study is. The key underlying issue here is that forced displacement is not typically a ‘single’ or ‘isolated’ treatment: displaced populations often face circumstances that may themselves lead to, or compound, the impacts of forced displacement per se, such as mental and physical harm from direct exposure to disasters or violence, as well as continued human rights violations before, during and after migration. For the choice of empirical strategy, studying the impact of displacement, or instead of exposure to a natural disaster or conflict violence, may require different approaches. In general terms, studies interested in the effect of exposure to conflict violence require the analyst to focus the empirical analyses on data for non-displaced populations. This is the case for existing studies examining birth outcomes (Fatušić *et al.*, 2005; Mansour and Rees, 2012; Valente, 2015) and infant mortality (Kudo, 2019), which focused on conflict violence impacts by analysing data for the general population that has been exposed to such violence over time, for instance in regions where the potential for conflict-induced displacement was limited (e.g. Mansour and Rees, 2012 examine data from the 2004 Demographic and Health Survey in Palestine during the al-Aqsa Intifada, when severe Israeli-imposed travel restrictions prevented significant out-migration from the West Bank). Instead, to isolate the effect of forced displacement, researchers usually need to compare individuals with similar exposure to the conflict or emergency event, some of whom remained in their home territory while others migrated. Yet individuals who decided to stay longer under an emergency are unlikely to be similar to those who fled earlier to other territories. Time of exposure and intensity of exposure to violence or disasters influence the probability of displacement, as well as people’s short- and long-term outcomes. The probability of displacement is also determined by factors like health status, wealth and social connections, among others that are often unobserved.

#### Data limitations

A key problem faced by researchers trying to effectively address the analytical challenges mentioned previously has been the scarcity of high-quality data. Ideally, analysts would require longitudinal data including relevant information before, during and after the emergency, for individuals who were exposed and not exposed to the emergency. This would, in principle, facilitate more reliable investigation of causal health impacts, although much depends of course on the possibility of capturing, through these longitudinal data, the contextual confounders discussed so far and that may be relevant in the setting of interest. However, humanitarian crises that produce large influxes of displaced populations are often unanticipated, making prospective data collection challenging. The movements of displaced groups are also difficult to predict and follow over time. Finally, comparable non-exposed groups may not be easily identified, since humanitarian crises frequently affect the vast majority of a population without strict boundaries. Such characteristics create enormous challenges for data collection efforts.

Due to these constraints, most studies of displacement effects are conducted using surveys or administrative data, covering short periods of time, for a few population groups, usually with no longitudinal follow-up allowing for panel data analyses. Omitted variables and measurement error then become frequent problems, as does the impossibility of adequately identifying forced migrants in the data, with a mixture of heterogeneous migrant groups in the analysis. Some studies have assumed that migrants from specific backgrounds (e.g. from the former Yugoslavia, see Janevic *et al.*, 2011), correspond to populations forcibly displaced by emergency events. Such assumptions could be inaccurate, introducing the risk of misclassification biases.

### Effect of what? The key role of the comparison groups

Based on the studies reviewed, we present a simple emerging taxonomy of possible comparison groups for applied research in the area, along with examples of research questions that can be investigated through such comparisons (Table 3). The groupings consider pairwise comparisons among three types of populations including the native population (neither migrant nor displaced), international (voluntary) migrants and forcibly displaced persons [including internally displaced persons (IDPs) and forced international migrants]. For these three population groups, comparisons between populations from a territory of origin (A) and a host territory (B) are possible, producing nine combinations. The two most frequent comparisons we identified in the literature are: (1) differences between international migrants from A relocated to B and the native population from B (type 4 comparison); (2) differences between forced international migrants from A relocated to B and the native population from B (type 7 comparison).

**Table 3.** A taxonomy of types of comparisons in migration studies

		Host territory (B)		
Comparison groups		Native population(non-displaced population)	International (voluntary) migrants	Forced displacement [internally displaced persons (IDPs)/forced international migrants]
Territory of origin (A)	Native population(non-displaced population)	1. Differences between native populations living in A and B (outside the migration literature)	2. Differences between native population of A and international migrants from A after relocation to B	3. Differences between native population of A and forced migrants from A after relocation to B
		*Example of research question: Cross-country comparisons of health conditions (unrelated to migration).*	*Example of research question: Health seeking behaviours of migrant and non-migrant populations from a common country of origin.*	*Example of research question: Health expenditures of forced migrants from a country compared to their populations of origin.*
	International (voluntary) migrants	4. Differences between international migrants from A relocated to B and the native population from B	5. Differences between international migrants from A relocated to B and international migrants from other territories (C) relocated to B	6. Differences between international migrants from A relocated to B and forced international migrants from other territories (C) relocated to B
		*Example of research question: Relative frequency of hospital admissions for a certain condition between international migrants and the native population in a territory.*	*Example of research question: Comparisons of mortality patterns between international migrants of different origins living in the same country.*	*Example of research question: Health expenditure of forced migrants from a country compared to their populations of origin.*
	Forced displacement (IDPs/forced international migrants)	7. Differences between forced international migrants from A relocated to B and the native population from B	8. Differences between forced international migrants from A relocated to B and international migrants from A relocated to B	9. Differences between forced international migrants from A relocated to B and forced international migrants from other territories (C) relocated to B
		*Example of research question: Access to health services by forced migrants/IDPs and native population groups.*	*Example of research question: Effect of having been forced to migrate on a health outcome.*	*Example of research question: Comparisons of disease patterns between forced migrants of different origins living in the same country.*

Source: authors’ elaboration.

While results from all these types of studies are sometimes presented as evidence about the impact of forced displacement on health, interpretation of causal inference in each of these designs is substantially different. One of the most frequent applications is the comparison of a health outcome, e.g. cause-specific mortality, between the native population of a host country and refugees (type 7 comparison) or international migrants (type 4 comparison) in that country (for examples, see Stewart *et al.*, 2008; Gagnon *et al.*, 2013). This type of research has led to the emergence of the ‘healthy migrant paradox’, whereby better health outcomes for various conditions have been observed among the migrant population when compared with the host population. This phenomenon can be explained in many contexts by the presence of selection bias, as younger, healthier and wealthier people are more likely to attempt to relocate and to overcome migration policies from host countries (Constant *et al.*, 2017). This is the case e.g. during protracted conflicts, where people who manage to survive and can make their way to other territories are more likely to be better-off to begin with. Such differences may be difficult to measure, particularly if important covariates are not directly comparable between groups. For example, the ‘quality’ of years of schooling in country A and country B may not be directly comparable as a measure of socioeconomic status or literacy, so that simply adjusting for years of education in a regression setting will not solve the problem of unbalanced characteristics between populations. Therefore, neither type 4 nor type 7 comparisons would produce unbiased estimates of the causal impact of displacement on health outcomes. These comparisons would be useful to characterize differences between groups, but not the make claims about the causes of such differences. Some studies have, however, undertaken type 7 comparisons focusing on IDP populations and having native populations as valid comparison groups; see e.g. Kuvacic *et al.*, 1996; Avogo and Agadjanian, 2010; Verwimp *et al.*, 2017.

Another possible approach to unearth causal links between forced displacement and health is to compare outcomes between forced migrants in a relocation territory and their non-displaced counterparts who remained in their home territory (type 3 comparison) (Singh *et al.*, 2004). While this design may be well suited to the task, it has limitations. Stayers could be individuals with fewer opportunities of free movement (e.g. sicker, older or poorer) or otherwise not directly affected by the emergency event. Careful selection of the control group to ensure comparability is then key in such settings. Availability of longitudinal individual data before and after the exogenous event could help overcome this selection bias issue. Alternatively, if feasible, researchers can use retrospective survey questions for the study population, gathering information about past events, health and other aspects prior to migration as baseline data (Brück *et al.*, 2016). Such an approach allows the analyst to account for the retrospective observable characteristics and time-invariant characteristics of individuals, but can introduce problems of recall bias, particularly for events that are not salient enough. Recall surveys also can only be applied to potentially selected samples of survivors if the interest is in one-off events like deaths. Even in these situations, type 3 comparisons can be useful to understand differences between individuals who were able to migrate during a protracted emergency and those staying in their home territory.

In some settings, it is possible to compare voluntary international migrants and forced migrants from a common origin who have relocated to the same host country (type 8 comparison). An example of this application is found in Avogo and Agadjanian (2010). This approach facilitates the adequate handling of baseline differences between migrant groups and is potentially the most suitable to produce unbiased displacement effect estimates in contexts where longitudinal individual data before and after displacement is not available. Time of arrival to the host country can be an important factor driving differences between groups however, due to integration and acculturation processes. An ideal comparison group would be a sample of voluntary migrants who relocated to the host country immediately before the (unanticipated) displacement emergency, for reasons unrelated to the event. A variation of the type 8 comparison that has been applied in some studies is the use of international migrants from another territory as the comparison group; see e.g. Charania *et al*. (2020) or Weeks and Rumbaut (1991).

### Effect on what? The main health outcomes of interest

The most frequent outcomes reported in the relevant literature are all-cause and cause-specific mortality (10 studies reviewed), both in the social sciences (Armenian *et al.*, 1993; DesMeules *et al.*, 2005; Montalvo and Reynal-Querol, 2007; Oostrum *et al.*, 2011; Hollander *et al.*, 2012; Saarela and Elo, 2016; Haukka *et al.*, 2017; Thordardottir *et al.*, 2020) and health sciences literature (Saarela and Finnas, 2009; Bauer *et al.*, 2019). Infant mortality (four studies: Weeks and Rumbaut, 1991; Avogo and Agadjanian, 2010; Oostrum *et al.*, 2011; Thordardottir *et al.*, 2020), maternal and perinatal outcomes (one systematic review study included, which identified 19 articles: see Harakow *et al.*, 2021), child morbidity (five studies: Bozzoli and Tilman, 2010; Lichtl *et al.*, 2017; Charania *et al.*, 2020; Thordardottir *et al.*, 2020; Hertting *et al.*, 2021) and child growth (four studies: Pak, 2010; Pak *et al.*, 2011; Schwekendiek and Pak, 2009; Sonne *et al.*, 2019) are the second most frequently studied groups of outcomes.

Less frequent have been studies among forcibly displaced populations focusing on maternal mortality (three studies: Janevic *et al.*, 2011; Oostrum *et al.*, 2011; Hynes *et al.*, 2012), self-perceived health (three studies: Chiswick *et al.*, 2008; Ruiz and Vargas-Silva, 2018; Zilic, 2018), unmet health needs (two studies: Gagnon *et al.*, 2007; 2013), access to services (three studies: Avogo and Agadjanian, 2010; Gagnon *et al.*, 2013; Small *et al.*, 2008), mental health (two studies: Stewart *et al.*, 2008; Tekeli-yesil *et al.*, 2018), fertility (one study: Verwimp *et al.*, 2017) and health-related behaviours (one study: Abdel-Rahim *et al.*, 2018).

Of the above studies, very few differentiated between short- and long-term effects of displacement on the relevant outcomes. Finally, we did not find studies analysing outcomes such as health-related quality of life or financial protection (e.g. total, out-of-pocket or catastrophic health expenditures).

### How to estimate health impacts? Main methods adopted to analyse causal links between forced displacement and health

Common empirical strategies adopted in the literature have been simple differences in the context of multivariable analysis, in which the displaced group is compared with a reference group (natives from the host country or other foreign-born individuals who did not suffer displacement) (e.g. Ruiz and Vargas-Silva, 2018), or simple tabular comparisons with or without formal statistical hypothesis testing. Those descriptive or exploratory studies have been particularly prevalent in health sciences, where most of the studies identified in systematic reviews report single group differences (e.g. before–after prevalence of symptoms or health conditions) or simple differences between two groups (Spiegel *et al.*, 2007). Double-difference approaches, such as difference-in-differences estimation, have been frequently used in the labour economics literature on forced displacement (e.g. Balkan *et al.*, 2018), yet rarely to study the impact on health outcomes. The only such example we could find is a study by Baez (2011), who uses double- and triple-differences specifications to examine short- and long-term health effects of forced migration on host communities.

Instrumental variables estimation has also been conducted in some studies^4^. While its application to the analysis of non-health impacts of displacement has been extensive, its use has been limited in the literature on health and forced migration. Notable exceptions are Bozzoli and Tilman (2010), Baez (2011), and more recently Zilic (2018) and Sonne *et al.* (2019). These studies rely on indices measuring the intensity of conflict, based on the distance from households to conflict episodes, or the number of civilian casualties per location, as the instrumental variable. Similar approaches have been increasingly used in recent years to study effects of displacement on the host population (see Verme and Schuettler, 2019 for details about the specific methods used).

Matching techniques have also been adopted to seek causal inference in displacement studies: an example is the use of propensity score matching to assess the probability of displacement in the context of war (Parpia and Khawaja, 2019). Yet we did not find displacement studies applying matching techniques with a focus on health outcomes. Similarly, we were unable to find studies using regression discontinuity designs, and although some studies had time-series data available for some health outcomes (e.g. Fatušić *et al.*, 2005), there is currently a lack of application also of methods that are appropriate for time-series analysis.

Parameter estimation has largely been conducted using ordinary least-squares, generalized linear models like probit/logit (for binary and categorical data) or Poisson/negative binomial (for count data like death rates). Probit has been favoured in economic or econometric analysis (Chiswick *et al.*, 2008; Bozzoli and Tilman, 2010; Baez, 2011; Giuntella and Mazzonna, 2015), while logit has been most frequently used in the health sciences (Gagnon *et al.*, 2007, 2013). We have also identified some examples of time-to-event (survival) models, usually with proportional hazard assumptions, including continuous-time (Cox models) (Thordardottir *et al.*, 2020) and discrete-time models (logistic regression) (Avogo and Agadjanian, 2010; Saarela and Elo, 2016), particularly to study mortality outcomes.

## Case study: the impact of forced displacement on reproductive health

In this section, we illustrate the challenges outlined above for identifying the causal health effects of displacement, as well as the implications of the methodological approaches selected to address these challenges, for the evidence-base on the broad domain of reproductive health.

### Characteristics of the studies

From 1454 articles identified, a total of 43 studies were reviewed that have addressed causal questions related to the impact of forced displacement on maternal and child health indicators. Populations of interest (‘treated groups’) included international forced migrants (*n* = 40) and IDPs (*n* = 4)^5^, described as refugees, asylum-seekers, displaced populations, returnees or migrants from different origins. Additionally, one study examined health impacts on host communities. The online supplementary material (SM2 and SM4) gives the number of studies included for each health indicator and an overview of the characteristics of the studies reviewed.

Data used includes household survey data, with individuals and mother–child dyads the most frequent units of observation. Retrospective cohorts through linkage of different administrative sources have been used in some studies analysing mortality data. Other studies have constructed cohort datasets based on cross-sectional data of retrospective obstetric histories. While repeated cross-sectional surveys were available in several contexts, the use of longitudinal data was infrequent, with only one study using panel data from a survey with several waves.

In nine studies, the territory of origin of displaced groups was unclear or not specified, with another five studies reporting origin as aggregate multinational areas (e.g. Balkans and Eastern Europe, Central/South America) or a non-exhaustive list of the countries of origin. African countries were the most frequent territories of origin of the forcibly displaced populations studied. Host territories were specified in all but one study. European countries were the most frequent host territories, followed by African countries.

Several studies used imprecise definitions of the population of interest. Some studies have defined population groups based on ethnicity, assuming that certain ethnic groups corresponded to refugees or asylum seekers (e.g. Schulpen *et al.*, 2001; Small *et al.*, 2008). Other studies, particularly for the USA, have focused on the legal status of migrants to define the comparison groups—e.g. assessing differences in birthweight among children of undocumented, documented immigrants and US-born Latinas (see Kelaher and Jones, 2002; Reed *et al.*, 2005)^6^. Most analyses used type 7 comparison groups. Only a few used alternative approaches such as comparing forced migrants to voluntary international migrants from countries of a similar ethnic background, same migrant populations before displacement, or close communities less or unaffected by the migration influx in cases of internal displacement (Avogo and Agadjanian, 2010; Baez, 2011; Verwimp *et al.*, 2017; Charania *et al.*, 2020; Thordardottir *et al.*, 2020).

Most of the studies reviewed used generalized linear model estimation methods with different distributions and link functions (probit, logit, binomial, Poisson and negative binomial regressions) or survival models (using parametric and semi-parametric methods). The second most frequent approach was the use of just descriptive tabulations or simple tests of differences in means, rates or proportions, dominant among less credible studies (see below). Fixed effects were frequently used to adjust for time-invariant unobserved characteristics of parents, households and/or geographical areas when survey data were available. Random effects by respondent or site were reported in a few cases, but the rationale for this decision and formal hypothesis testing comparing fixed and random effects was often absent. Time-fixed effects were rarely included, even in studies with multiple observation periods. In the case of survival models, proportional or identical death hazards over time were usually assumed, yet once again the rationale for such decisions was missing in most studies.

### What can we learn from the available evidence?

Quality appraisal for each study reviewed can be found in Table 4. Overall, the quality of reviewed studies was low, with the evidence from most of these classified as less credible. Strong empirical identification strategies were rare and, in almost all studies, the (often implicit) assumption of random allocation of displaced status between treated and potential control groups was unlikely to hold. The lack of an adequately justified and valid control group, along with severe limitations of the statistical methods applied, were the main reasons for most available studies being categorized as less credible. In at least one of the less credible studies, the data available suggest that a stronger methodology was possible for inference (e.g. difference-in-differences), since information on the outcome was available for forced migrants and non-displaced populations before and after the displacement event.

**Table 4.** Effects of displacement on reproductive health based on the credibility of the findings

Outcomes	Effect	*Strongly credible*	*Somewhat credible*	*Less credible*
Neonatal and perinatal mortality	↑			Kuvacic *et al.*, 1996; Schulpen, *et al.*, 2001; Råssjö *et al.*, 2013; Belihu *et al*., 2016; Bozorgmehr, 2018; Wanigaratne, 2018; Ozel *et al.*, 2018; Liu *et al.*, 2019
	↓			
	0			Small *et al.*, 2008; Oostrum *et al.*, 2011; Erenel *et al.*, 2017; Kanmaz; 2019
Child (<5 years old) mortality	↑	Baez, 2011	Avogo and Agadjanian, 2010	Singh *et al.*, 2004
	↓			
	0			Khawaja, 2004
Infant (<1 year old) mortality	↑			
	↓			Weeks and Rumbaut, 1991
	0			Oostrum *et al.*, 2011
<20 years old mortality	↑			
	↓		Thordardottir *et al.*, 2020 (suicide, Cardiovascular disease)	
	0		Thordardottir *et al.*, 2020 (all-cause)	
Child morbidity	↑	Bozzoli and Tilman, 2010; Baez, 2011	Thordardottir *et al.*, 2020	Lichtl *et al.*, 2017; Charania *et al.*, 2020; Hertting *et al.*, 2021
	↓			
	0			
Child growth	↑			
	↓	Baez, 2011		Schwekendiek and Pak, 2009; Pak, 2010
	0			
Maternal mortality	↑			Oostrum *et al.*, 2011
	↓			Hynes *et al.*, 2012
	0			
Maternal morbidity	↑			Stewart *et al.*, 2008; Hanegem *et al.*, 2011
	↓			
	0			
Caesarean delivery	↑			Small *et al.*, 2008; Kandasamy, 2014
	↓			Gagnon, 2013
	0			
Low birthweight delivery	↑			Weeks and Rumbaut, 1991; Kuvacic *et al.*, 1996
	↓			Small *et al.*, 2008; Janevic *et al.*, 2011
	0			
Preterm delivery	↑			Kuvacic *et al.*, 1996; Ozel *et al.*, 2018; Kanmaz, 2019;
	↓			Small *et al.*, 2008; Janevic *et al.*, 2011; Wanigaratne, 2018; Khan, 2018; Liu *et al.*, 2019; Agbemenu, 2019
	0			Kandasamy, 2014; Alnuaimi, 2017; Erenel *et al.*, 2017; Michaan, 2014
Access to services	↑			
	↓		Avogo and Agadjanian, 2010; Gagnon *et al.*, 2013	Small *et al.*, 2008
	0		Gagnon *et al.*, 2007	
Induced abortion	↑			Goosen, 2009 (rate)
	↓			Goosen, 2009 (ratio from pregnancies)
	0			

Note: ‘0’ includes null findings (statistically insignificant results).

Two studies were considered strongly credible, having in common the use of instrumental variables (Bozzoli and Tilman, 2010; Baez, 2011). Four other studies were considered somewhat credible based on including potentially valid comparison groups and some degree of control for endogeneity in the analysis (Avogo and Agadjanian, 2010; Gagnon *et al.*, 2007, 2013; Thordardottir *et al.*, 2020). A common characteristic of these credible studies was the explicit and narrow definition of their research questions concerning the displacement exposure and context being analysed, which provided crucial leads for selecting the appropriate data and methods for the analysis. For example, Baez (2011) defined forced displacement as the massive influx into the Tanzanian Kagera region, in early 1994, of refugees fleeing the genocides in Burundi and Rwanda. The study then investigated, as main research questions, the short- and long-run consequences of this forced displacement shock for anthropometric (height-for-age Z-score), morbidity (diarrhoea, fever) and infant mortality outcomes of the local Tanzanian children (i.e. children from the host population). Longitudinal survey data, analysed through an instrumental variable strategy, was then a natural approach to be selected in this setting, where displacement represented a sudden shock and where topographic barriers introduced variation in refugee intensity within the study region. Similarly, for investigating the research question of the impacts of forced displacement on child morbidity (acute illnesses in children <5 years old), Bozzoli and Tilman (2010) operationalized forced displacement as the particular situation of residency in IDP camps in Northern Uganda, following the government-mandated displacement of almost all residents of three districts of that conflict-affected region into such camps by 2005. This definition guided the collection of data to permit the application of an instrumental variable estimation approach based on the characteristics of such a displacement process, involving geo-coded information about conflict intensity at the place of birth of the head of household.

The evidence on the topic is thin in general, as implied by the scarcity of credible studies, and there remain important uncertainties. For the three outcomes for which strongly credible evidence is available, lower quality studies agree with the direction of estimated effects. This is the case for child mortality, morbidity and child growth, for which findings are consistent, suggesting that forced displacement increases under-5’s mortality and disease risk (particularly infectious diseases) and negatively affects child growth. As an example, the available evidence indicates that under-5’s mortality nearly doubles in the years immediately after migration among forcibly displaced children (Avogo and Agadjanian, 2010) and increases by ∼10% in communities receiving a large influx of displaced individuals (Baez, 2011).

It is noteworthy that even in the absence of strongly credible evidence, various less credible studies report an increased risk of neonatal and perinatal deaths after forced displacement. This is particularly clear for stillbirths, with several studies suggesting at least a 2-fold increase (e.g. Råssjö *et al.*, 2013; Belihu *et al.*, 2016; Erenel *et al.*, 2017; Ozel *et al.*, 2018; Liu *et al.*, 2019). These studies usually focus on forcibly displaced populations that have recently migrated, potentially capturing the *in utero* effects of the different shocks experienced before and during the process of forced displacement.

A few studies suggest that fertility rates increase in the short-term for forced migrants, who also appear to experience worse access to maternal and childhood services, even in the presence of greater need. The most common limitation of these studies has been to perform comparisons of forcibly displaced groups with the native host population, where other factors beyond displacement are likely to explain (at least partially) the observed differences in outcomes.

Finally, contradictory findings are the rule for several outcomes for which the evidence is less credible, such as maternal mortality, low birthweight, prematurity and caesarean section delivery. The case of prematurity is particularly salient: the 13 studies that examine this outcome report great heterogeneity in findings, including about the direction of displacement impacts, making it impossible to establish any firm conclusions. An important reason for such heterogeneity is the often very broad research questions posed, particularly around what displacement exposure is being analysed, making it difficult to compare findings across studies and resulting in unclear directions about data and methods that could be appropriate to answer the study questions. For example, Ozel *et al*. (2018) focus on the obstetric outcomes among Syrian refugees who gave birth in a tertiary hospital in Turkey, in 2015, compared to the same outcomes among ethnic Turkish women. There are several potential sources of heterogeneity within this displaced group (Syrian refugees), including health conditions in the community of origin, circumstances, reasons for and time since displacement, given that the Syrian civil war began in 2011. Unfortunately, information about many of these confounders which could influence outcomes in this broad displacement context was not available for the simple outcome comparisons undertaken by the authors, limiting also what we can learn about the consequences of different displacement processes (e.g. short-term and long-term).

## Discussion and conclusions

Our study reviews the main challenges faced by applied researchers to produce unbiased causal estimates of the effects of forced displacement on health. Some of these challenges are present more generally in any applied causal inference research that relies on observational data, including non-random allocation of the ‘treatment’ or exposure of interest, difficulties in identifying proper control groups, data constraints and limited comparability of exposure events. Some challenges are unique to the analysis of forced-migration impacts, such as the close link between exposure to conflict/violence and features of the displacement process, making it difficult to disentangle the causal effects of each one, and more generally the many potential drivers of changes in health that arise within such a socially complex phenomenon as forced displacement.

We provide an emerging taxonomy of potential comparison groups based on the existing literature in the field. The most frequently used comparison group was the native population from the host community. In most studies, this comparison group arguably did not represent a valid control group for robust inference about the causal effect of forced displacement on health. Therefore, most of these existing effect estimates are likely affected by substantial bias. Younger, healthier and wealthier persons are more likely to migrate, or migrate sooner, in a context of crisis. Additionally, host communities tend to be better off in many aspects that influence health status (e.g. regarding healthcare, education, income) than the populations of origin of forced migrants. These contextual differences introduce endogeneity and have often confounded the reported links between forced displacement and health outcomes.

Overall, high-quality causal inference methods were rarely found in the literature analysing the health effects of forced displacement. We frequently found non-comparative or simple difference studies that, unfortunately, offer little or no information about the research question of what the causal health effects of forced displacement are. More credible empirical strategies were found mainly in studies analysing effects on mortality within natural experiments (e.g. Ceded Karelia during WWII; Saarela and Elo, 2016; Saarela and Finnas, 2009). In our case study on reproductive health, we identified only two studies with strongly credible designs (Bozzoli and Tilman, 2010; Baez, 2011), which reported negative effects on child mortality and morbidity. Although few in number, those studies demonstrate that it is possible to obtain robust causal inference on the topic through judicious non-experimental data analysis. A common aspect of their approaches is to exploit features of the particular emergency setting to generate instrumental variables (e.g. conflict intensity indices) that can predict the ‘treatment’ variable (e.g. conflict-induced displacement), but are arguably uncorrelated with the health outcomes of interest or with other unobservable individual/contextual factors that may influence who is ‘treated’. We must emphasize that the prevalence of instrumental variables approaches in the credible studies reviewed here should not be understood as pointing to a ‘correct’ causal inference approach in every analytical setting. Instead, the best method to be adopted (difference-in-differences, interrupted time series, instrumental variables etc.) will invariably depend on the specific question, context and data at hand.

Beyond these few credible studies, most available evidence on a wide range of health outcomes reports estimates that are prone to substantial bias, making it difficult to draw any reliable conclusions that may help guide policy action, for instance. Health domains for which more and better evidence about forced displacement impacts is needed include mental health, unmet health needs, health behaviours and broad reproductive health outcomes such as maternal mortality, low birthweight and prematurity. Further research is urgently needed in these domains to reduce the uncertainty in findings, through empirical design strategies that include the selection of valid comparison groups and control of potential endogeneity through robust estimation methods, guided by well-defined research questions and displacement contexts.

Based on the results of our review, there are some key take-home messages to improve future applied research in the area and reduce existing knowledge gaps.

First, the selection of a valid control group is an essential methodological step, yet it is too often overlooked. In settings of internal or cross-border displacement to neighbouring countries, valid control groups may be found in geographically close communities with similar baseline characteristics and who were arguably unaffected by the exogenous event that triggered displacement. In international displacement settings, comparisons with international (voluntary) migrants from a similar ethnic background (ideally from the same location), who relocated to the same host country as the displaced communities, is likely to be the best possible approach. In all cases, the similarities and differences between the displaced population and the comparison group should be properly scrutinized, discussed and addressed. Gathering data—and providing the rationale for its use—to ensure comparability of confounding factors that could explain variation in health outcomes is essential. The use of matching techniques may allow the researcher to address many of these confounding issues, at least with respect to observable confounders. As such, more frequent implementation of matching methods seems warranted.

Second, since in most cases phenomena like exposure to man-made emergencies, natural disasters and ensuing mass displacements can only be studied using observational data, more robust findings will require a wider (and, of course, judicious) adoption of non-experimental methods better suited for causal inference, such as instrumental variables, difference-in-differences, regression discontinuity and interrupted time series analyses. Most of these methods are familiar to researchers trained in econometrics, epidemiology and statistics. While in some situations it may be challenging to identify natural experiments, or the available data simply may not be good enough to facilitate the use of any of these approaches, we found evidence that such stronger analytical approaches could have been applied in several studies reviewed. This implies that it is indeed feasible for researchers to strengthen the current knowledge base on the health-related consequences of forced displacement by making better use of non-experimental methods and data to provide more credible causal insights. While in many instances the insights generated may be of more immediate relevance for the specific displacement context under study, useful information for other research settings will probably be generated, such as about promising methods, data or particular health impacts to be investigated further. Where new data collection in protracted emergencies and displacement settings is feasible, researchers should carefully consider what/how data need to be collected in order to enable application of high-quality inferential approaches. This includes, e.g. ensuring the comparability of new measurement tools with previous surveys or administrative data collected for the population of interest before the emergency event occurred.

Another overall message from our review is the great importance, for both the analytical choices and usefulness of studies about forced-displacement impacts, of starting with a clear definition of what the ‘displacement treatment’ under evaluation is or, more generally, of what the research question is concerning forced displacement and in what context such ‘treatment’ has taken place (e.g. sudden mass displacement due to a natural disaster vs a protracted situation of human rights violations among a population that shares a specific characteristic, such as ethnicity, leading to the displacement of that same population in a phased manner over time). We discuss examples in our reproductive health case study that illustrate where broad (or not explicit) definitions of displacement exposure in some studies were also linked to less robust analyses and conclusions, and (conversely) examples where well-defined research questions and displacement context were conducive to more robust analyses. Explicitly and carefully defining the displacement ‘treatment’ and context is crucial also to understand the potential generalization of conclusions from one study to other settings, i.e. their external validity.

We must note four main limitations of our study. First, we highlighted that a key contribution of our paper is to use a systematic and extensive approach to identify and assess the relevant literature on forced migration from a broad disciplinary perspective. Nevertheless, due to the rapid growth of this literature, it is possible that we have missed relevant studies in the field, particularly those available as working papers or grey literature not easily accessible from health and social sciences search engines.

Second, synthetizing the evidence about the health impacts of forced displacement through a scoping or systematic review exercise presents enormous challenges, essentially due to the inherently multidimensional, socially complex nature of the forced-displacement phenomenon. We have previously noted how some of the literature reviewed in our study adopted definitions of the forced displacement ‘treatment’ (or its context), population of interest and associated research questions, that were very broad or not explicitly outlined. This is in contrast with the smaller set of most robust studies identified in our case study, which had the common feature of explicitly stating well-defined research questions, the specific forced-displacement context and of which populations. The differences we have identified in contextual clarity and comparability across the forced-displacement literature as a whole limit what we can feasibly learn from scoping reviews like our own, highlighting the value of further reviews in the future that focus more narrowly on studies addressing questions such as the health effects of specific types of forced displacement (see e.g. Table 3) in a particular setting (e.g. civil conflict or natural disaster).

Third (and linked to the previous issue), although we believe that our scoping review makes an important contribution by facilitating a comprehensive synthesis of different types of studies across different disciplines, there is an inherent limit to the translation of learnings about the health effects of forced displacement from one setting to another, chiefly due to the many different contextual characteristics of forced displacement in any given setting. Yet we believe that our categorization of study types according to evidence strength, discussion of data and methodological options for applied researchers, along with the resulting recommendations of good practice arising from this scoping review, do provide an important contribution for improvements in the evidence base on the topic, including by offering guidance to promote the external validity of findings from future applied studies.

A final limitation of our study is that we have focused our analysis on quantitative studies addressing causal questions. Yet qualitative research has an important role to help understand patterns and pathways that could explain quantitative findings, generate hypotheses to guide further research enquiry and to study questions and outcomes that are difficult to investigate using quantitative instruments like surveys. While excluding qualitative studies from our review was justified based on the aim of our research, qualitative methods can provide very important contextual information to help understand the findings arising from the literature on the impacts of forced displacement, calling for wider interdisciplinary collaborations to advance future research in the field.

## Supplementary Material

czad002_Supp

## Endnotes

1. Endogeneity, in econometric terminology, arises when an explanatory variable is correlated with the error term. It refers to the situation when the exposure or treatment of interest (*X*) is explained by a third variable (*Z*) that is also related to the outcome of interest (*Y*). *Z* in this case is an endogenous variable. This phenomenon can occur due to omitted variables, measurement error or simultaneity. The term endogeneity is closely related to the concept of confounding in the epidemiological and statistical literature. Details about these concepts can be found elsewhere (Gunasekara *et al.*, 2008).

2. The term empirical strategy refers to the overall process of approaching a research question and the application of methods to address it, including defining the causal effect of interest and the assumptions required to answer the question with the available data.

3. In our context, exogenous events refer to external influences that are arguably unpredicted and beyond the control of the individuals, forcing a population to migrate. Examples of such exogenous events may be natural disasters and armed conflicts.

4. An instrumental variable (IV) is a third variable *Z* that, in addition to the outcome variable (*Y*) and the exposure (or treatment) variable of interest (*X*), is used to produce unbiased regression estimates in settings where *X* is likely correlated with the error term due to endogeneity. In other words, *Z* is used to control for unobserved confounders. More information on IV methods can be found elsewhere (Baiocchi *et al.*, 2014).

5. One study included both forced international migrants and IDPs.

6. This type of study does not allow the analyst to draw robust conclusions about the health effects that are attributable to forced displacement specifically, rather than to the undocumented migrant status. Therefore, we excluded studies using this design from our case study.

## Reflexivity statement

The group of authors includes researchers at the early (M.L.), mid- (C.C.) and senior (R.M.-S.) career stages, from South America (Chile) and Europe (UK). This represents a good balance in terms of seniority. Although we were not able to achieve gender balance in the authorship of this manuscript (three males), we note that the work in this article is part of a suite of research pieces that are currently being undertaken under a wider research project (see Acknowledgements) where the majority of co-investigators are females (7 out of 12 co-investigators), who are also the main authors of most project outputs. However, within the project, the set of specialist skills essential to the undertaking of the specific research in this manuscript was only available from the listed authors.

## Reason why all authors are from high-income countries

This manuscript is the result of a desk review of literature spanning all world regions and countries where relevant literature could be identified. There was no fieldwork conducted in any country, and the design of the research, data analysis and interpretation, and drafting of the article were undertaken by the listed authors.
