# Supplementary material for: What is the impact of forced displacement on health? A scoping review
Source: Health Policy Plan. 2023 Jan 11;38(3):394–408. doi: 10.1093/heapol/czad002 (PMC10019572; doi:10.1093/heapol/czad002)
Supplement: czad002_Supp [file czad002_supp.zip › Supplementary Material_RR.docx]

## Supplementary Material

**SM1. Full electronic search strategy**

Epistemonikos

(title:((title:(refugee) OR abstract:(refugee)) OR (title:(refugees) OR abstract:(refugees)) OR (title:(asylum) OR abstract:(asylum)) OR (title:(internally displaced persons) OR abstract:(internally displaced persons))) OR abstract:((title:(refugee) OR abstract:(refugee)) OR (title:(refugees) OR abstract:(refugees)) OR (title:(asylum) OR abstract:(asylum)) OR (title:(internally displaced persons) OR abstract:(internally displaced persons))))

Econlit

(refugee OR refugees OR asylum OR displace* OR (forced AND migra*)) AND health

EMBASE

1 'refugee'/exp

2 'asylum'/exp

3 ' protracted displacement'

4 'internally displaced persons'/exp

5 health

6 caus*

7 1 or 2 or 3 or 4

8 5 and 6 and 7

**SM2. PRISMA flow diagram**

**Identification of studies via databases and registers**

Records removed *before screening*:

Duplicate records removed (n = 56)

Records marked as ineligible by automation tools (n = 0)

Records removed for other reasons (n = 0)

Records identified from databases (n = 1454)

**Identification**

Records screened

(n = 1398)

Records excluded

(n = 1211)

Reports excluded* (n=131):

Non-comparative studies (n= 70)

Unclear if displacement is the main exposure variable (n = 59)

No health outcome reported (n=2)

No full text available (n = 0)

**Screening**

Reports assessed for eligibility

(n = 187)

Primary studies included by outcome category**:

Maternal and perinatal outcomes (n=19)

All-cause mortality (n=10)

Child growth (n=4)

Infant mortality (n=4)

Child morbidity (n=5)

Self-perceived health (n=3)

Maternal mortality (n=3)

Access to services (n=3)

Other (n=6) (e.g. mental health, unmet health needs, fertility, health-related behaviors)

Primary studies included in the review (n = 56)

**Included**

Primary studies on reproductive health (maternal, perinatal and child health) (n=43)

* Reports can have multiple causes for exclusion but the first criteria identified is recorded.

** Studies do not sum up because some included studies reported outcomes from multiple categories.

*Based on:*  Page MJ, McKenzie JE, Bossuyt PM, Boutron I, Hoffmann TC, Mulrow CD, et al. The PRISMA 2020 statement: an updated guideline for reporting systematic reviews. BMJ 2021;372:n71. doi: 10.1136/bmj.n71

For more information, visit: <http://www.prisma-statement.org/>

**SM3. Data extraction form**

| Data dimension | Explanation |
| --- | --- |
| First author | First author |
| Title | Title of the article |
| Year | Year of publication |
| Journal | Journal where article was published |
| Comparative study | Used for screening: (1) single group; (2) comparative (at least two groups) |
| Outcome category | Broad categories of main outcomes such as infant mortality; maternal mortality; child growth; child morbidity; mental health; perinatal outcomes; among others |
| Type of effects | Short-term, less than 10 years from exposure; vs long-term; more than 10 years from exposure |
| Type of migration (IM or IDP) | International migrant (IM) or internally displaced population (IDP) |
| Type of comparison | 1 to 9, based on table 3 (main text) |
| Origin territory | Territory where forced migrants come from |
| Host territory | Territory where forced migrants arrive |
| Exposed group | Group in which the exposure is studied (treated) |
| Control group(s) | Group(s) used as comparators |
| Data collection | Type of study including cohorts (retrospective, prospective), cross-sectional (single, repeated) and data source (administrative or survey) |
| Number of time points | For cohorts or repeated cross-sectional, number of years or waves |
| Number of sites | Number of sites involved in the analysis (locations, countries or hospital depending on study design) |
| Location | Sites where data was collected |
| Sample size | Number of participants |
| Statistical method | Main methods |
| Outcomes | List of primary and secondary outcomes reported |
| Results | Main results |
| Quality | Quality appraisal based on table 2 (main text) |

**SM4. Overview of study characteristics**

| First author | Year | Outcome categ. | Type of migration | Type of comparison | Origin territory | Host territory | Exposed group | Control group(s) | Data collection | Location | Sample size | Outcomes | Quality |
| --- | --- | --- | --- | --- | --- | --- | --- | --- | --- | --- | --- | --- | --- |
| Agbemenu | 2019 | Perinatal outcomes | IM | 7 (refugees vs host country - USA) | African | USA | Refugee | Native women | Cohort | USA | 77891 | Preterm birth | Less credible |
| Alnuaimi | 2017 | Perinatal outcomes | IM | 7 (refugees vs host country - Jordan) | Syria | Jordan | Refugee | Native women | Retrospective cohort | Jordan | 1260 | Preterm birth | Less credible |
| ﻿Avogo | 2010 | Infant mortality | IDP | 3 (War-displaced vs migrants before displacement to Luanda in a given year), 7 (War-displaced arrived to Luanda vs non-migrants from Luanda), 8 (War-displaced vs non-war migrants arrived to Luanda) | Angola (other areas; non-specified) | Angola (Luanda) | War-migrants | a) Non-war migrants; b) Permanent residents (native population); c) Non-migrants in a given year | Cross-sectional (survey) | Samba and Viana (Greater Luanda) | 719 | a) Under-five year infant mortality; b) Number of antenatal visits; c) Child delivery in a health facility; d) Child full immunization at 1-year | Somewhat credible |
| Badshah | 2011 | Perinatal outcomes | IM | 7 (refugees vs host country - Pakistan) | Different countries | Pakistan | Refugee | Native women | Unclear | Pakistan | 1039 | a) Spontaneous abortion; b) Preterm birth | Less credible |
| Baez | 2011 | Infant mortality; child growth; child morbidity | IM | Not applicable | Burundi and Rwanda | Tanzania (Kagera) | Refugees (genocide) | Low influx areas in Tanzania | Cross-sectional (pooled data from two surveys) / longitudinal survey (5 waves) | Tanzania (Kagera, Mwanza and Mara regions) / Tanzania (Kagera) | 500 - 2000 (depending on the model) | a) ﻿Under five infant mortality; b) child's anthropometrics (z-scores of height for age; height in early childhood); c) morbidity (probability of have diarrhoea or fever in the last two-weeks); d) Schooling; e) Prevalence of chronic disease in early adulthood | Strongly credible |
| Belihu | 2016 | Perinatal outcomes | IM | 7 (refugees vs host country - Australia) | East-African countries | Australia | Refugee | Native women | Cross-sectional study | Australia | 432567 | a) Perinatal mortality; b) Preterm birth | Less credible |
| Bozorgmehr | 2018 | Perinatal outcomes; maternal morbidity | IM | 7 (refugees vs host country - Germany) | Different countries | Germany | Asylum-seeking women | Native women | Cross-sectional study | Germany | 19864 | a) High risk pregancy disease; b) Spontaneous abortion; c) Preterm birth | Less credible |
| Bozzoli | 2010 | Child morbidity | IDP |  | Uganda | Uganda | IDP in refugee camps | IDP returnees | Cross-sectional (survey) | Northern Uganda (Amuru, Gulu, Kitgum, Pader, Lira and Oyam) | 3962 households | a) Symptoms of acute illness in the last two weeks in children U5 (﻿malaria/fever, diarrhoea and severe cough) | Strongly credible |
| ﻿Charania | 2020 | Child morbidity | IM | 7 (refugees from different backgrounds vs NZ non-migrant children) 8 (migrant children from different background vs NZ non-migrant children) 10 (refugees from different backgrounds vs migrant children from different background) | Not specified | New Zealand | Refugees | Non-migrant NZ chilldren (host territory) | Cohort (NZ multiple administrative sources) | New Zealand | 692919 | a) Vaccine‐Preventable Disease‐Associated Hospitalisations | Less credible |
| Demirci | 2016 | Maternal morbidity | IM | 7 (refugees vs host country - Turkey) | Different countries | Turkey | Refugee | Native women | Retrospective cohort | Turkey | 1090 | Preeclampsia | Less credible |
| Erenel | 2017 | Maternal morbidity | IM | 7 (refugees vs host country - Turkey) | Different countries | Turkey | Refugee | Native women | Retrospective cohort | Turkey | 600 | a) Preeclampsia; b) Stilbirth; c) Preterm birth | Less credible |
| Gagnon | 2007 | Unmeet need | IM | 7 (refugees vs host country - Canadian) 7 (refugees-claimants vs host country - Canadian) 4 (international migrants vs host country - Canadian) | Unclear | Canada | Refugees and asylum seekers | Host population (Canada); non-refugee international migrants (different backgrounds) | Cross-sectional (survey and home visit 7-10 days after delivery) | Canada | 341 | a) Post-partum concerns not addressed by the health system by the children and mother (long list) | Somewhat credible |
| Gagnon | 2013(a) | Unmeet need | IM | 7 (refugees vs host country - Canadian) 7 (refugees-claimants vs host country - Canadian) 4 (international migrants vs host country - Canadian) | Unclear | Canada | Refugees and asylum seekers | Host population (Canada); non-refugee international migrants (different backgrounds) | Cross-sectional (survey and home visit 1 week and 4-months after delivery) | Canada | 1127 | a) Post-partum concerns not addressed by the health system by the children and mother (long list) | Somewhat credible |
| Gagnon | 2013(b) | Mode of delivery | IM | 7 (refugees vs other immigrants (non refugee / AS)) 7 (asylum-seeker vs other immigrants (non refugee / AS)) | Unlear (different backgrounds) | Canada | Refugees; asylum seeker | Other (non refugee / AS) immigrants | Cohort | Canada (Toronto, Montreal, Vancouver) | 1025 | Emergency cesarean section | Less credible |
| Goosen | 2009 | Abortion; Fertility | IM | 7 (asylum seekers vs host country - Netherlands) | Different countries (e.g. Congo, Angola, Somalia, Sudan, Azerbaijan, Former Yugoslavia, Afghanistan, Iraq, China, Sri Lanka) | Netherlands | Asylum seekers | Host population (Netherlands) | Cross-sectional (administrative data) | Netherlands | 3347596 | a) induced abortion rate; b) induced abortion ratio; c) teenage birth rate | Less credible |
| Güngör | 2018 | Maternal morbidity | IM | 7 (refugees vs host country - Turkey) | Unclear | Turkey | Refugee | Native women | Cross-sectional study | Turkey | 1484 | a) Preeclampsia; b) Maternal infections | Less credible |
| Hertting | 2021 | Child morbidity | IM | 7 (asyllum seekers vs host country - Sweden, includes migrants non asyllum seekers) | Unclear | Sweden | Asylum seekers | Host population (Sweden) | Cross-section (hospital data) | Sweden | 342138 | a) Hospital discharge due to an infection diagnosis | Less credible |
| Hynes | 2012 | Maternal mortality | IM | 7 (refugees vs host country population, for 10 countries) | 10 countries (Bangladesh, Chad, Ethiopia, Kenya, Nepal, Rwanda, Sudan, Tanzania, Uganda, Zambia) | Unclear | Refugee mothers from 10 countries (Bangladesh, Chad, Ethiopia, Kenya, Nepal, Rwanda, Sudan, Tanzania, Uganda, Zambia) | Host populations (not clearly explained)+ | Surveys and refugee camp reports | Unclear | 110248 | a) Maternal mortality ratio | Less credible |
| Kandasamy | 2014 | Perinatal outcomes; mode of delivery | IM | 7 (refugees vs host country - Canadian) | Unclear (different backgrounds) | Canada | Refugees | Host population (Canada) | Cohort (administrative) | Canada (Toronto) | 547 | a) low birth weight; b) mode of delivery | Less credible |
| Kanmaz | 2019 | Perinatal outcomes; maternal morbidity | IM | 7 (refugees vs host country - Turkey) | Syria | Turkey | Refugee | Native women | Retrospective cohort | Turkey | 17000 | a) Preeclampsia; b) Stillbirth; c) Preterm birth | Less credible |
| Khan | 2017 | Maternal morbidity | IM | 7 (refugees vs host country - Canada) | Different countries | Canada | Refugee | Native women; non-refugee migrant | Population-based cohort | Canada | 4092 | a) Preeclampsia; b) Preterm birth | Less credible |
| Khawaja | 2004 | Infant mortality | IM | 7 (Refugees vs host country - Jordan and Lebanon) | Palestine | Jordan; Lebanon | Refugees (suposedly all Palestinians, it is unclear if refugees of other origings are also present in Jordan and Lebanon camps) | Refugees in Palestine; Non-refugees in Palestine, Lebanon and Jordan | Cross-sectional (surveys) | Palestine, Jordan and Lebanon | 124292 | a) Infant mortality rates (IMR); b) Under five years mortality rates (U5MR) | Less credible |
| Kuvacic | 1996 | Perinatal outcomes | IDP and IM | 7 (IDP (Croatia, occupied areas) vs non-displaced population, Croatia (Zagreb)) 7 (Refugees (Bosnia) vs non-displaced population, Croatia (Zagreb)) 3 (IDP (Croatia, occupied areas) in the post-war vs same population in the pre-war period) | Croatia, Bosnia and Herzegobina | Croatia (Zagreb) | IDP from occupied areas of Croatia and refugees from Bosnia and Herzegovina | Non-displaced Croatian population | Repeated cross-sectional | Croatia | 9150 | Premature deliveries, low birth weight, perinatal outcome (Apgar and mortality) | Less credible |
| Lichtl | 2017 | Child morbidity | IM | 7 (Asylum-seekers vs host country - Germany, includes migrants) | Unclear | Germany (Baden-Wurttemberg) | Asylum seekers | Host population | Cross-sectional (administrative hospital records) | Germany (Baden-Wurttemberg) | 21742 | a) Ambulatory care sensitive hospitalizations (1) Allergies & allergic re- actions, 2) Asthma, 3) Convulsions, 4) Dental condi- tions, 5) Diabetes mellitus, 6) Failure to thrive, 7) Gastritis, 8) Gastroenteritis / dehydration, 9) Immunization-preventable diseases, 10) Inflammatory diseases of female pelvic organs, 11) Iron deficiency anaemia / anaemia, 12) Kidney- and urinary infections, 13) Nutritional deficiency, 14) Neonatal jaundice, 15) Severe ENT- infection 16) Skin infection, 17) Doctor’s orders have not been followed by patient) | Less credible (comparison based on host population does not allow for a valid comparison group) |
| Liu | 2019 | Perinatal outcomes; maternal morbidity | IM | 7 (refugees vs host country - Sweden) | Different countries | Sweden | Refugee | Native women | Register-based cohort | Sweden | 31897 | a) Preeclampsia; b) Stillbirth; c) Preterm birth | Less credible |
| Michaan | 2014 | Perinatal outcomes | IM | 7 (refugees vs host country - Israel) | Eritrea and Sudan | Israel | Refugee | Native women | Retrospective cohort | Israel | 494 | Preterm birth | Less credible |
| Miller | 2016 | Perinatal outcomes | IM | 7 (refugees vs host country - USA) | Unclear | Syracuse, NY, USA | Refugee | Native women | Cohort | Syracuse, NY, USA | 6354 | Preterm birth | Less credible |
| Nilsen | 2018 | Maternal morbidity | IM | 7 (refugees vs host country - Norway) | Different countries | Norway | Refugee | Native women | Cross-sectional study | Norway | 1287270 | Preeclampsia | Less credible |
| Ozel | 2018 | Perinatal outcomes; maternal morbidity | IM | 7 (refugees vs host country - Turkey) | Unclear | Turkey | Refugee | Native women | Retrospective cohort | Turkey | 1152 | a) Preeclampsia; b) Stillbirth; c) Preterm birth; d) PPROM | Less credible |
| Pak | 2010 | Child growth | IM | 7 (refugees vs host country - South Korea) | North Korea | South Korea | Refugees | Host population (South Korea) | Cross-sectional (survey) | South Korea | 1406 | a) Height-for-age; b) weight-for-height; c) z-score height; d) z-score weight; e) BMI | Less credible |
| Rässjö | 2013 | Perinatal outcomes; maternal morbidity | IM | 7 (refugees vs host country - Sweden) | Somalia | Sweden | Refugee | Native women | Case-control study | Sweden | 524 | a) Preeclampsia; b) Stillbirth; c) Preterm birth | Less credible |
| ﻿Schwekendiek | 2009 | Child growth | IM | 7 (refugees vs host country - South Korea) | North Korea | South Korea | Refugees | Host population (South Korea) | Cross-sectional (survey) | South Korea |  | a) Weight; b) Height; c) BMI | Less credible |
| Shears | 1987 | Morbidiity | IM |  |  |  |  |  |  |  |  |  |  |
| Singh | 2004 | Infant mortality | IM | No formal comparison | Arua District (Uganda), Yei River District (Sudan) | Arua District (Uganda), Yei River District (Sudan) | Displaced population (including returnees) | a) Stayees; b) Displaced at different ages (<15 yrs; >15yrs); returnees at different ages (<15 yrs; >15yrs) | Cross-sectional (survey) | Arua District (Uganda), Yei River District (Sudan) | 2504 | a) Under five mortality | Less credible |
| Stewart | 2008 | Mental health | IM | 7 (refugees vs host country - Canadian) 7 (asylum seekers vs host country - Canadian) 4 (international migrants vs host country - Canadian) | Unclear | Canada | Refugees and asylum seekers | Host population (Canada); non-refugee international migrants (different backgrounds) | Cross-sectional (surveys) | Canada (Montreal, Toronto, Vancouver) | 341 | a) Postpartum depression | Somewhat credible |
| Thordardottir | 2020 | Infant mortality | IM | 10, especial case of 4 (Balkan war-displaced migrants vs other European countries arrived to Sweden) | Balkan countries (Albania, Bosnia-Herzegovina, Croatia, Macedonia, Slovenia, or Yugoslavia) | Sweden | Migrants from Balkan countries entering Sweden between 1991-2001 (assumed forced displaced) | Migrants from other European countries entering Sweden (Austria, Belgium, Czech Republic, Czechoslovakia, Denmark, Finland, France, Germany, Great Britain, Greece, Hungary, Iceland, Ireland, Italy, Malta, Moldavia, the Netherlands, Norway, Poland, Portugal, Romania, Slovak Republic, Spain, or Switzerland) | Cohort (Swedish registry) | Sweden | 252166 | a) Disease-specific mortality; b) Disease-specific incidence | Somewhat credible |
| Van Hanegem | 2011 | Maternal morbidity | IM | 7 (asylum seekers vs host country - Netherlands) | Different countries | Netherlands | Asylum seekers | Host population (Netherlands); Non-western non-refugee migrants (multiple origins) | Cohort | Netherlands | 432043 | Severe acute maternal morbidity (SAMM) defined as: a) ICU admission; b) uterine rupture; c) Eclampsia/HELLP; d) major obstetric haemorrhage; e) others. | Less credible |
| van Oostrum | 2011 | Maternal mortality | IM | 7 (refugees vs host country - Netherlands) | Different countries (e.g. Congo, Angola, Somalia, Sudan, Azerbaijan, Former Yugoslavia, Afghanistan, Iraq, China, Sri Lanka) | Netherlands | Asylum seekers | Host population (Netherlands) | Cross-sectional (administrative data for 2002-2005) | Netherlands | 222217 | a) Maternal mortality ratio; b) Perinatal mortality; c) Infant mortality | Less credible |
| ﻿Verwimp | 2017 | Fertility | IDP | 7 (IDP vs non-displaced population) | Burundi | Burundi | Refugees | Non displaced population | Cross-sectional (survey) | Burundi | 4783 | a) Fertility | Strongly credible |
| Wanigaratne | 2018 | Perinatal outcomes; maternal morbidity |  | 7 (refugees vs host country - Canada) | Different countries | Canada | Refugee | Native women; non-refugee migrant | Population-based cohort | Canada | 893066 | a) Preeclampsia; b) Stillbirth; c) Preterm birth; d) HIV | Less credible |
| Weeks | 1991 | Infant mortality | IM | 8 (Indochinese refugees vs other non-refugee migrants (Other Asian, Hispanic)) 7 (Indochinese refugees vs other ethnic no migrants (Black, Non Hispanic White)) | Indochinese (Vietnam, Laos and Cambodia) | USA (San Diego) | Indochinese (Vietnam, Laos and Cambodia) refugees | US population from different ethnic backgrounds | Cohort (administrative data from different sources) | US | 269252 | a) Infant mortality; b) Birthweight; c) Time of onset of prenatal care | Less credible |

**SM5. Preferred Reporting Items for Systematic reviews and Meta-Analyses extension for Scoping Reviews (PRISMA-ScR) Checklist**

| **SECTION** | **ITEM** | **PRISMA-ScR CHECKLIST ITEM** | **REPORTED ON PAGE #** |
| --- | --- | --- | --- |
| **TITLE** | | | |
| Title | 1 | Identify the report as a scoping review. | Page 1 |
| **ABSTRACT** | | | |
| Structured summary | 2 | Provide a structured summary that includes (as applicable): background, objectives, eligibility criteria, sources of evidence, charting methods, results, and conclusions that relate to the review questions and objectives. | Abstract provided following the journal guidelines (Page 1) |
| **INTRODUCTION** | | | |
| Rationale | 3 | Describe the rationale for the review in the context of what is already known. Explain why the review questions/objectives lend themselves to a scoping review approach. | Pages 6-7 |
| Objectives | 4 | Provide an explicit statement of the questions and objectives being addressed with reference to their key elements (e.g., population or participants, concepts, and context) or other relevant key elements used to conceptualize the review questions and/or objectives. | Page 7 |
| **METHODS** | | | |
| Protocol and registration | 5 | Indicate whether a review protocol exists; state if and where it can be accessed (e.g., a Web address); and if available, provide registration information, including the registration number. | No review protocol is available |
| Eligibility criteria | 6 | Specify characteristics of the sources of evidence used as eligibility criteria (e.g., years considered, language, and publication status), and provide a rationale. | Pages 7-8 and Supplementary Material-SM1 |
| Information sources* | 7 | Describe all information sources in the search (e.g., databases with dates of coverage and contact with authors to identify additional sources), as well as the date the most recent search was executed. | Page 7 and Supplementary Material-SM1 |
| Search | 8 | Present the full electronic search strategy for at least 1 database, including any limits used, such that it could be repeated. | Supplementary Material-SM1 |
| Selection of sources of evidence† | 9 | State the process for selecting sources of evidence (i.e., screening and eligibility) included in the scoping review. | Pages 8-9 |
| Data charting process‡ | 10 | Describe the methods of charting data from the included sources of evidence (e.g., calibrated forms or forms that have been tested by the team before their use, and whether data charting was done independently or in duplicate) and any processes for obtaining and confirming data from investigators. | Page 9 and Supplementary Material-SM3 |
| Data items | 11 | List and define all variables for which data were sought and any assumptions and simplifications made. | Page 9 and Supplementary Material-SM3 |
| Critical appraisal of individual sources of evidence§ | 12 | If done, provide a rationale for conducting a critical appraisal of included sources of evidence; describe the methods used and how this information was used in any data synthesis (if appropriate). | Page 9 and Table 2 |
| Synthesis of results | 13 | Describe the methods of handling and summarizing the data that were charted. | Pages 10-11 |
| **RESULTS** | | | |
| Selection of sources of evidence | 14 | Give numbers of sources of evidence screened, assessed for eligibility, and included in the review, with reasons for exclusions at each stage, ideally using a flow diagram. | Supplementary Material-SM2 and Pages 22-23 (reproductive health case study) |
| Characteristics of sources of evidence | 15 | For each source of evidence, present characteristics for which data were charted and provide the citations. | Supplementary Material-SM4 (reproductive health case study) and References |
| Critical appraisal within sources of evidence | 16 | If done, present data on critical appraisal of included sources of evidence (see item 12). | Sections 3-4, Table 4 and Supplementary Material-SM4 (reproductive health case study) |
| Results of individual sources of evidence | 17 | For each included source of evidence, present the relevant data that were charted that relate to the review questions and objectives. | Table 4 and Supplementary Material-SM4 (reproductive health case study) |
| Synthesis of results | 18 | Summarize and/or present the charting results as they relate to the review questions and objectives. | Sections 3-4, Tables 3-4, Supplementary Material-SM4 (reproductive health case study) |
| **DISCUSSION** | | | |
| Summary of evidence | 19 | Summarize the main results (including an overview of concepts, themes, and types of evidence available), link to the review questions and objectives, and consider the relevance to key groups. | Sections 3-4, Tables 3-4 |
| Limitations | 20 | Discuss the limitations of the scoping review process. | Pages 30-31 |
| Conclusions | 21 | Provide a general interpretation of the results with respect to the review questions and objectives, as well as potential implications and/or next steps. | Section 5 |
| **FUNDING** | | | |
| Funding | 22 | Describe sources of funding for the included sources of evidence, as well as sources of funding for the scoping review. Describe the role of the funders of the scoping review. | Description provided in separate Title Page as per the journal’s guidelines |

JBI = Joanna Briggs Institute; PRISMA-ScR = Preferred Reporting Items for Systematic reviews and Meta-Analyses extension for Scoping Reviews.

* Where *sources of evidence* (see second footnote) are compiled from, such as bibliographic databases, social media platforms, and Web sites.

† A more inclusive/heterogeneous term used to account for the different types of evidence or data sources (e.g., quantitative and/or qualitative research, expert opinion, and policy documents) that may be eligible in a scoping review as opposed to only studies. This is not to be confused with *information sources* (see first footnote).

‡ The frameworks by Arksey and O’Malley (6) and Levac and colleagues (7) and the JBI guidance (4, 5) refer to the process of data extraction in a scoping review as data charting*.*

§ The process of systematically examining research evidence to assess its validity, results, and relevance before using it to inform a decision. This term is used for items 12 and 19 instead of "risk of bias" (which is more applicable to systematic reviews of interventions) to include and acknowledge the various sources of evidence that may be used in a scoping review (e.g., quantitative and/or qualitative research, expert opinion, and policy document).

*From:* Tricco AC, Lillie E, Zarin W, O'Brien KK, Colquhoun H, Levac D, et al. PRISMA Extension for Scoping Reviews (PRISMAScR): Checklist and Explanation. Ann Intern Med. 2018;169:467–473. [doi: 10.7326/M18-0850](http://annals.org/aim/fullarticle/2700389/prisma-extension-scoping-reviews-prisma-scr-checklist-explanation).
